# Supplementary material for: Biallelic mutations in calcium release activated channel regulator 2A (CRACR2A) cause a primary immunodeficiency disorder
Source: eLife. 2021 Dec 15;10:e72559. doi: 10.7554/eLife.72559 (PMC8673834; doi:10.7554/eLife.72559)
Supplement: Supplementary file 1. [file elife-72559-supp1.docx]

**SUPPLEMENTARY MATERIALS**

**Supplementary Table 1. List of all the sgRNAs, and primers used for cDNA sub cloning and qRT-PCR in this study.**

| **Clone name** | **Forward Primer** | **Reverse Primer** | **Comments** |
| --- | --- | --- | --- |
| hCRACR2A-a_pLentiguide_sg1 | CAC CGA TCG CGA TGG CTG CCC CTG A | AAA CTC AGG GGC AGC CAT CGC GAT C | sgRNA targeting human CRACR2A |
| hCRACR2A-a_pLentiguide_sg2 | CAC CGC AAG GGC TTC ATC GCC AGG A | AAA CTC CTG GCG ATG AAG CCC TTG C | sgRNA targeting human CRACR2A |
| hCRACR2A-a_pLentiguide_sg3 | CAC CGG GGC GAC ATG GGC GAA GAT G | AAA CCA TCT TCG CCC ATG TCG CCC C | sgRNA targeting human CRACR2A |
| FG11F CRACR2A^E278D^ | TGT AAG GAG CAG GAG CTG GAT CAG CTC ACC CAG AAG CAG A | TCT GCT TCT GGG TGA GCT GAT CCA GCT CCT GCT CCT TAC A | Site directed mutagenesis (SDM) using wildtype CRACR2A (isoform a) cDNA as PCR template |
| FG11F CRACR2A^R144G^ | GAG AAG GTG TAT CTG TCC GGA GGG GAT GAG GAT CTG GGC | GCC CAG ATC CTC ATC CCC TCC GGA CAG ATA CAC CTT CTC | Site directed mutagenesis (SDM) using wildtype CRACR2A (isoform a) cDNA as PCR template |
| FG11F CRACR2A^R144G, E300*^ | CTG CAT CAT GAC AAG CAT TAG ACC AAG GCC GAA AAC ACA | TGT GTT TTC GGC CTT GGT CTA ATG CTT GTC ATG ATG CAG | Site directed mutagenesis (SDM) using FG11F CRACR2A^R144G^ as PCR template |
| hCRACR2A-a (isoform a) | AGA GAT CTG GCT CTG TGA TAG GCA | TCT TCA ACG GAG ATG ATT CTG CGG | qPCR primers |
| hIFN-γ | TTC AGC TCT GCA TCG TTT TG | TTA CTG GGA TGC TCT TCG AC | qPCR primers |
| hIL-2 | AGA ACT CAA ACC TCT GGA GGA AG | GCT GTC TCA TCA GCA TAT TCA CAC | qPCR primers |
| hTNF | CGA GTG ACA AGC CTG TAG C | GGT GTG GGT GAG GAG CAC AT | qPCR primers |
| h36B4 | AGATGCAGCAGATCCGCAT | GTTCTTGCCCATCAGCACC | qPCR primers |
