# Supplementary figures and images for: Biallelic mutations in calcium release activated channel regulator 2A (CRACR2A) cause a primary immunodeficiency disorder
Source: eLife. 2021 Dec 15;10:e72559. doi: 10.7554/eLife.72559 (PMC8673834; doi:10.7554/eLife.72559)

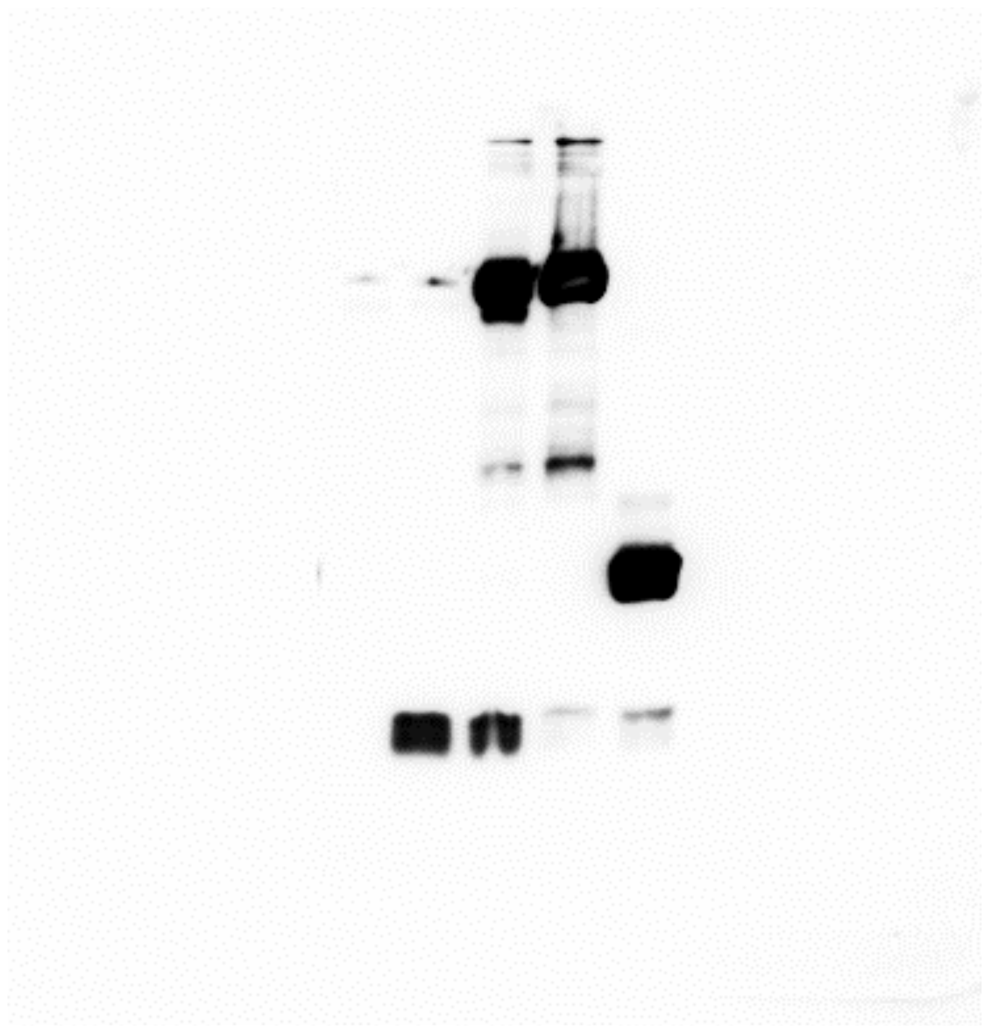

Supplement: Source data 1. — Zip folder containing raw and uncropped images for Western blots and Excel spreadsheets of quantitation. [file elife-72559-supp2.zip › 20210812_CRACR2A_SourceData/Figure 7B_Flag_right.tif]

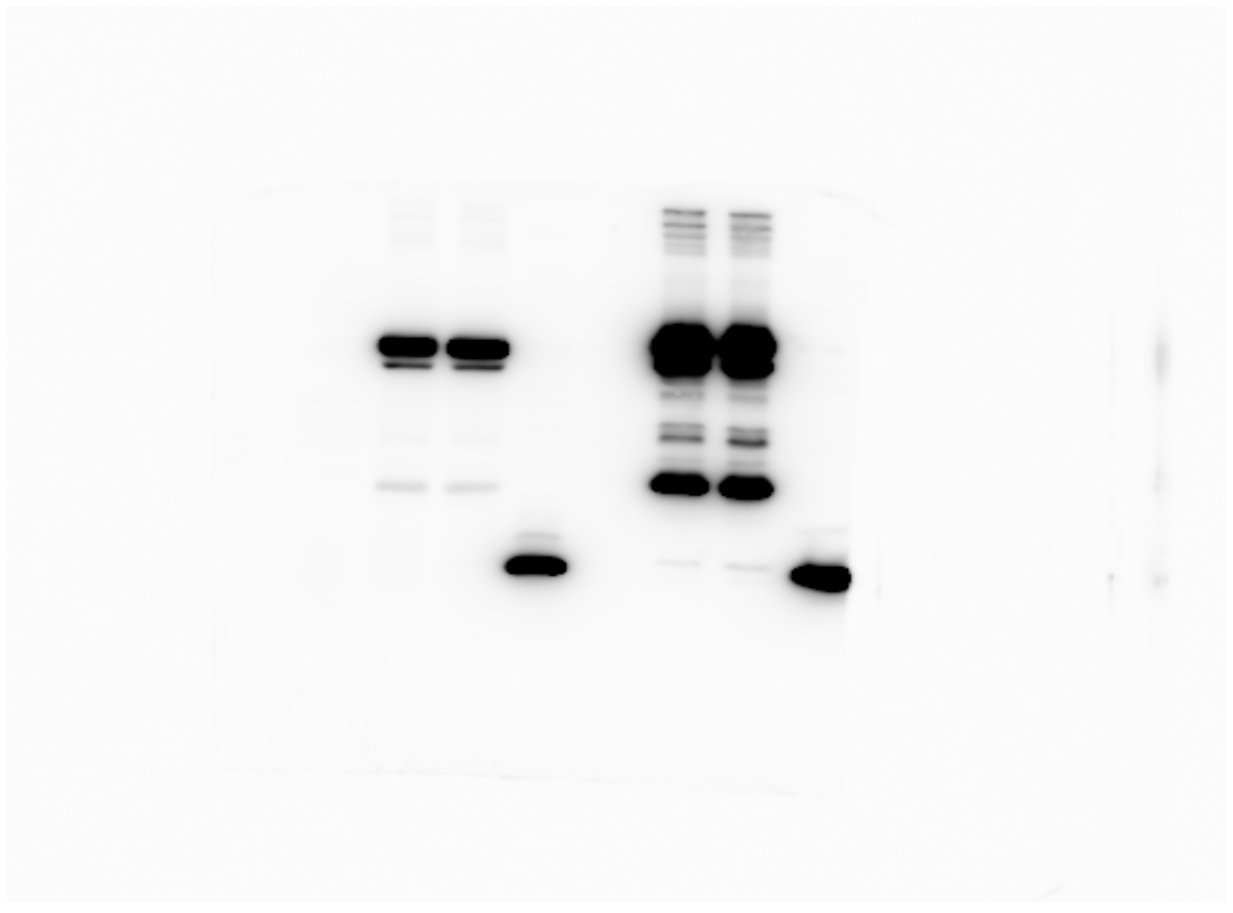

Supplement: Source data 1. — Zip folder containing raw and uncropped images for Western blots and Excel spreadsheets of quantitation. [file elife-72559-supp2.zip › 20210812_CRACR2A_SourceData/Figure 7A_Flag.tif]

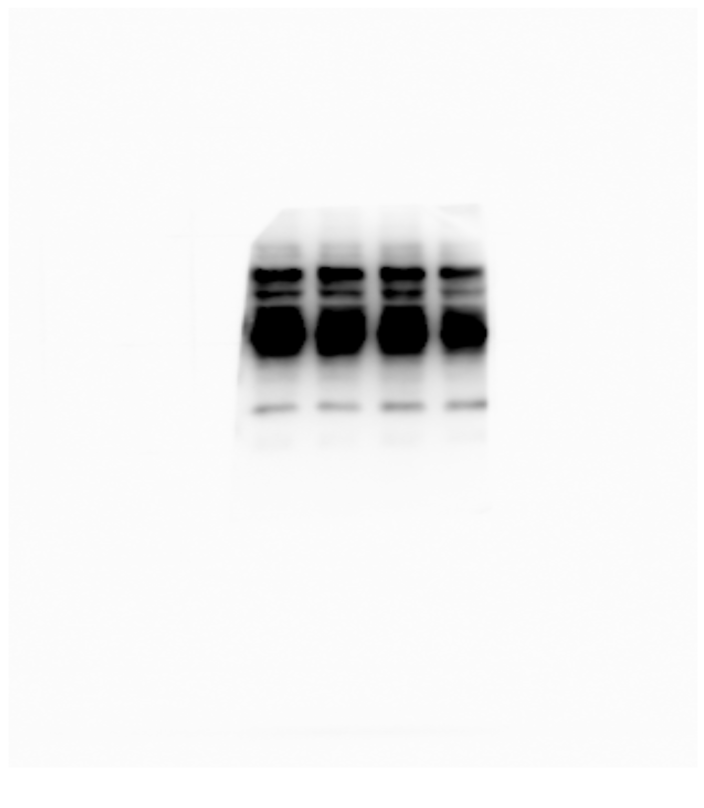

Supplement: Source data 1. — Zip folder containing raw and uncropped images for Western blots and Excel spreadsheets of quantitation. [file elife-72559-supp2.zip › 20210812_CRACR2A_SourceData/Figure 7A_Orai1-His_left.tif]

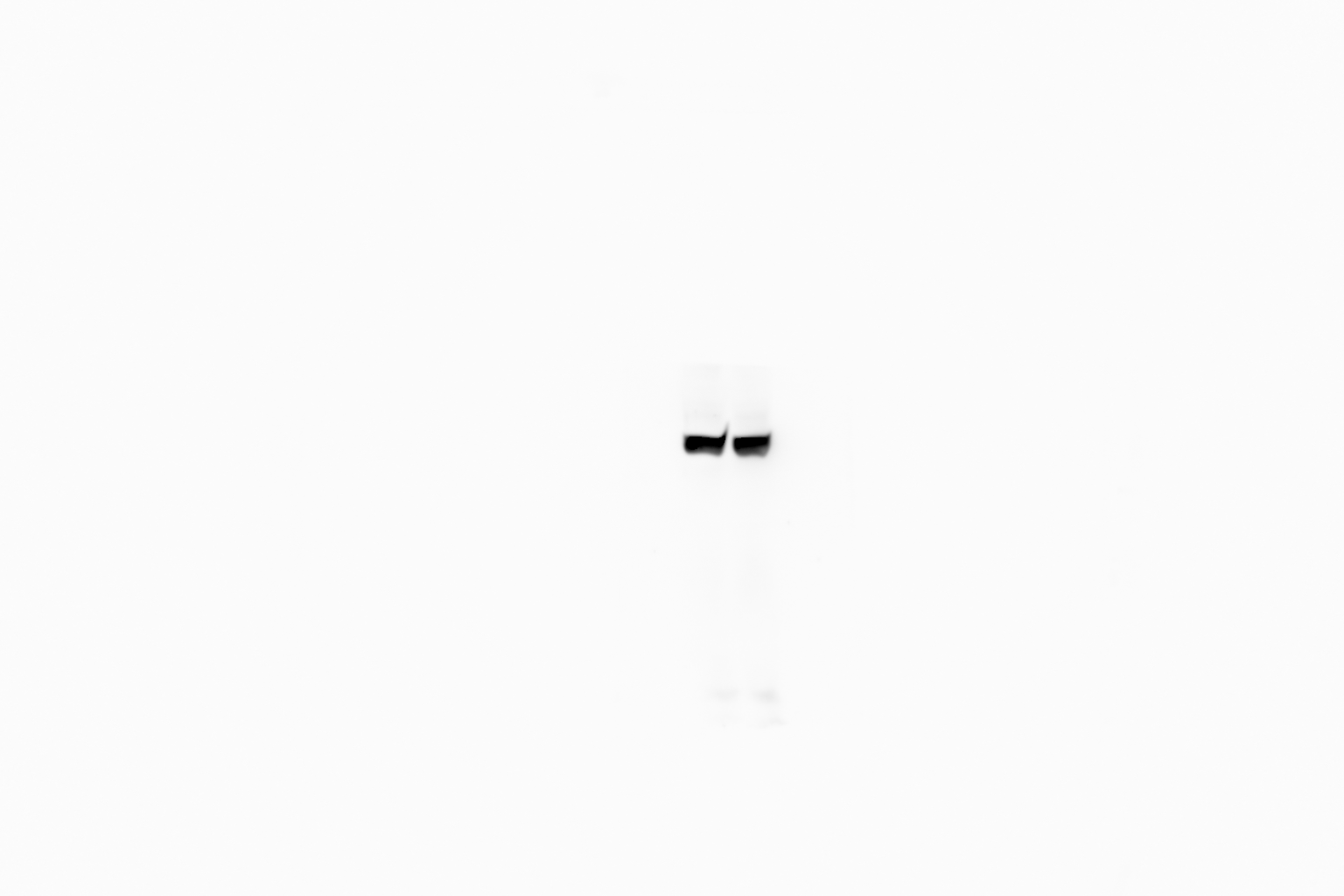

Supplement: Source data 1. — Zip folder containing raw and uncropped images for Western blots and Excel spreadsheets of quantitation. [file elife-72559-supp2.zip › 20210812_CRACR2A_SourceData/Figure 4_figure supplemnet 1_ORAI1_WB.tif]

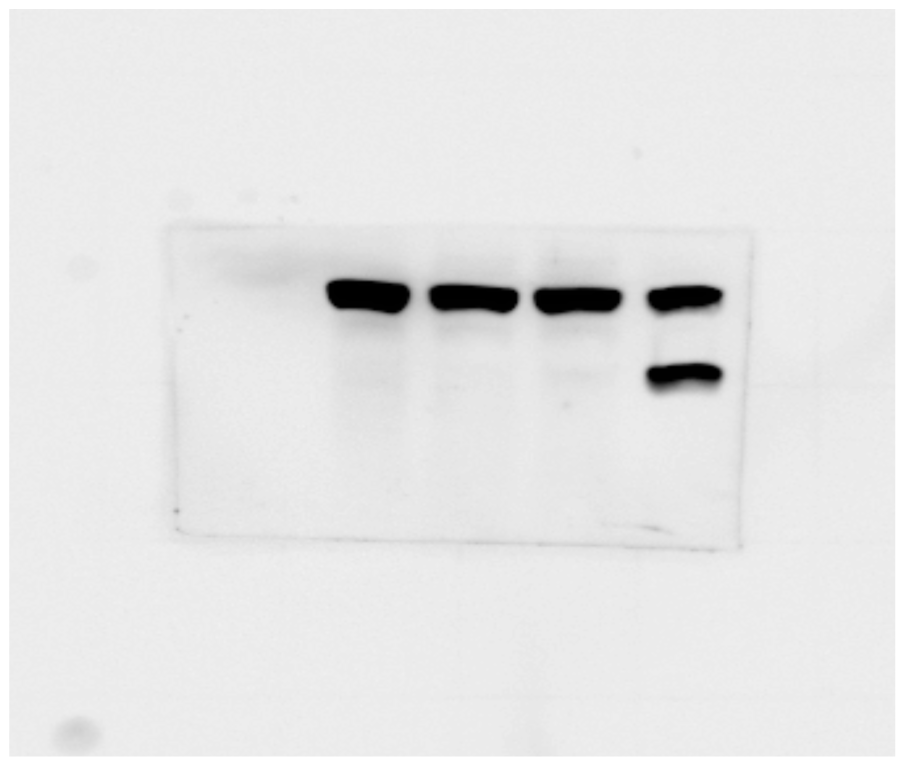

Supplement: Source data 1. — Zip folder containing raw and uncropped images for Western blots and Excel spreadsheets of quantitation. [file elife-72559-supp2.zip › 20210812_CRACR2A_SourceData/Figure 5_figure supplement 1_actin.tif]

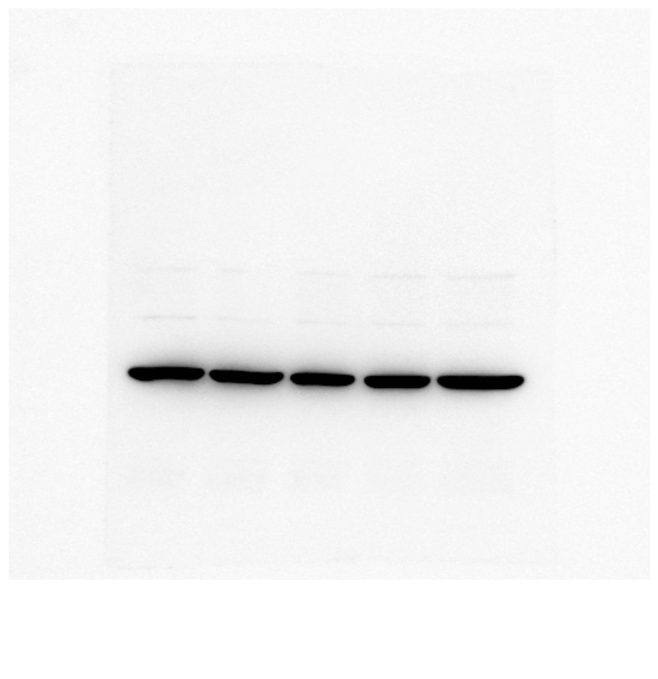

Supplement: Source data 1. — Zip folder containing raw and uncropped images for Western blots and Excel spreadsheets of quantitation. [file elife-72559-supp2.zip › 20210812_CRACR2A_SourceData/Figure 5_figure supplemnet_1C_actin.tif]

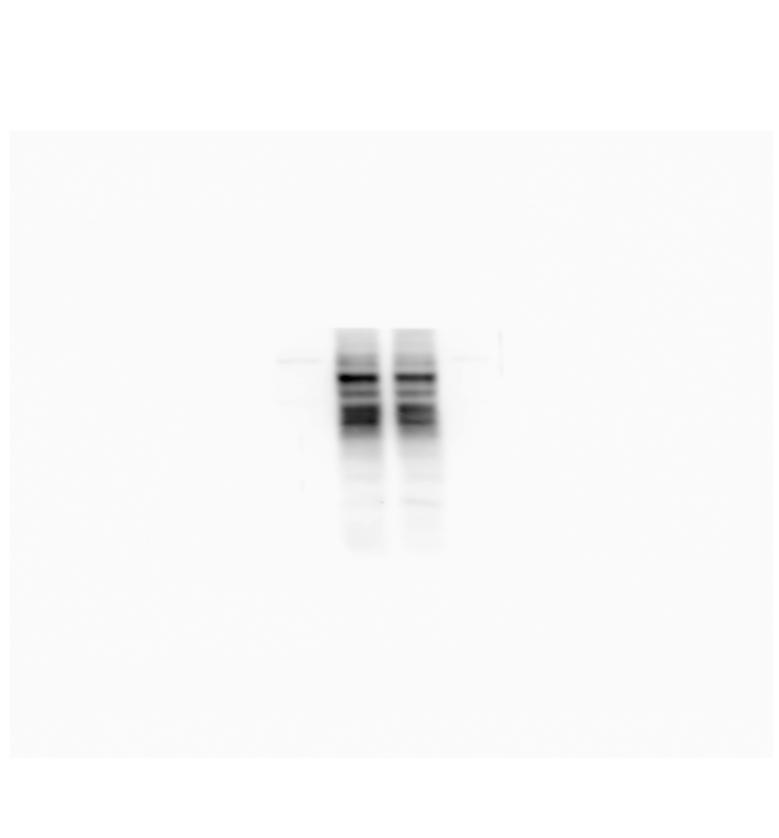

Supplement: Source data 1. — Zip folder containing raw and uncropped images for Western blots and Excel spreadsheets of quantitation. [file elife-72559-supp2.zip › 20210812_CRACR2A_SourceData/Figure 7A_Orai1-His_Right.tif]

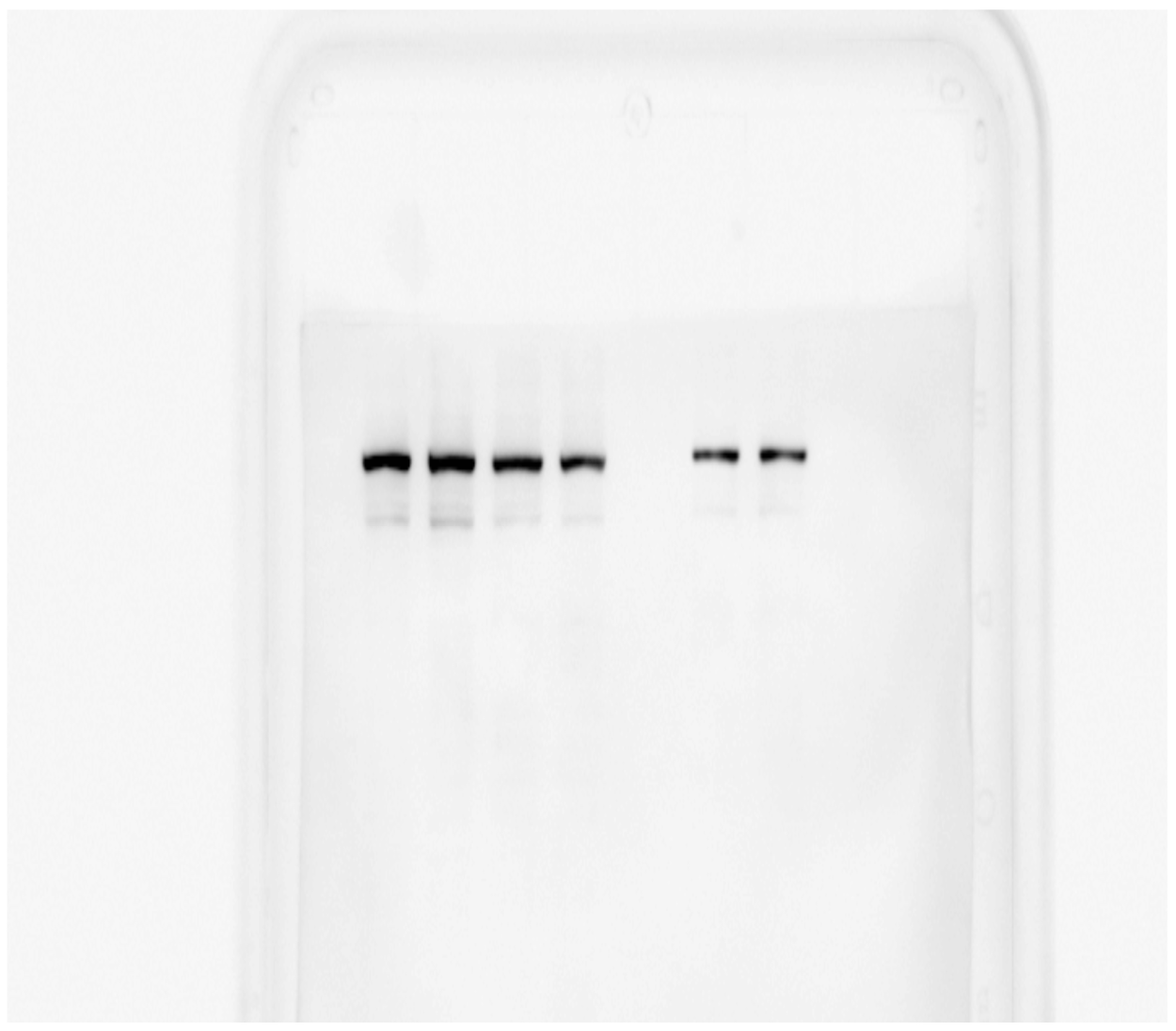

Supplement: Source data 1. — Zip folder containing raw and uncropped images for Western blots and Excel spreadsheets of quantitation. [file elife-72559-supp2.zip › 20210812_CRACR2A_SourceData/Figure 7B_GFP.tif]

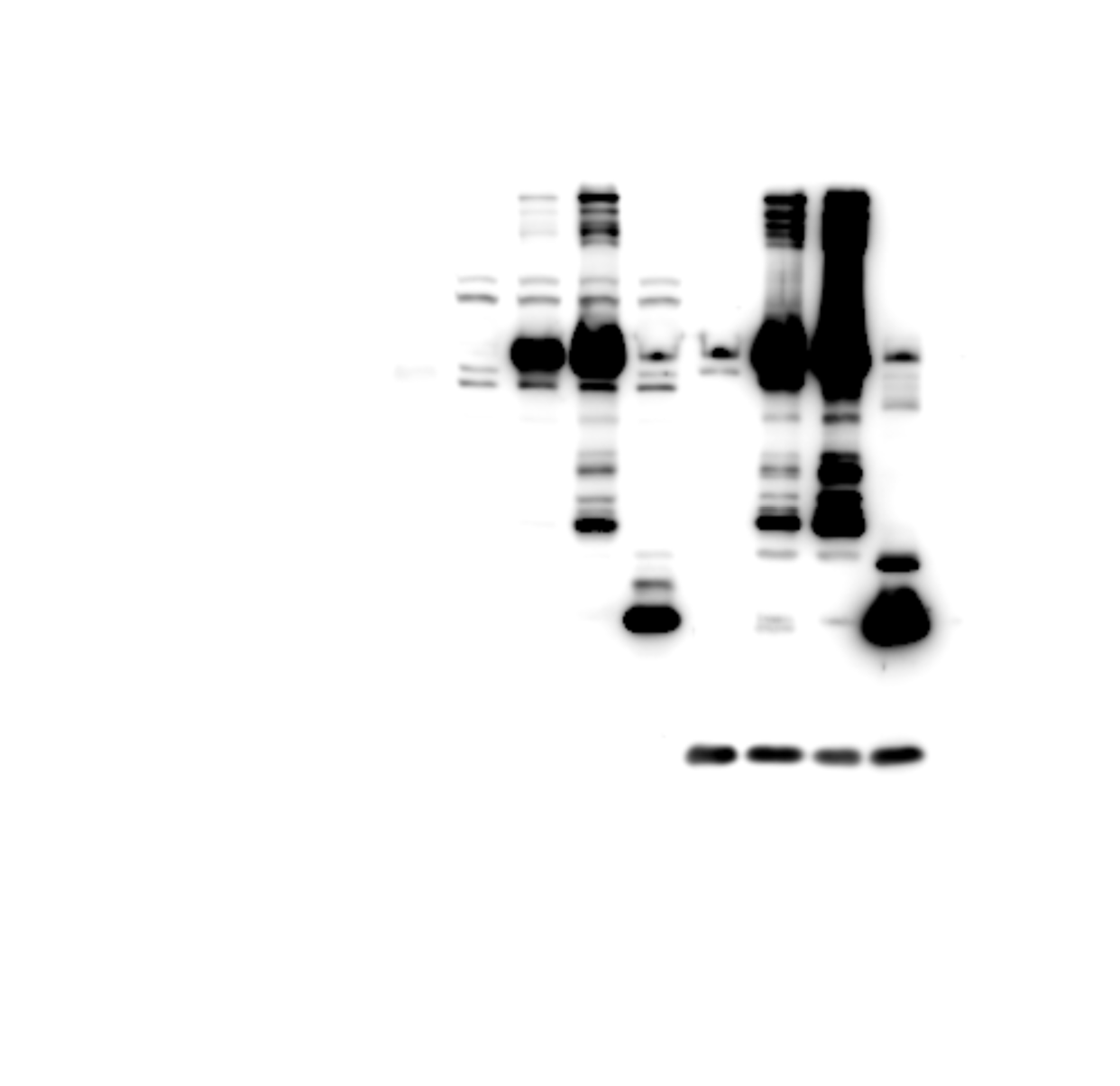

Supplement: Source data 1. — Zip folder containing raw and uncropped images for Western blots and Excel spreadsheets of quantitation. [file elife-72559-supp2.zip › 20210812_CRACR2A_SourceData/Figure 7B_Flag_left.tif]

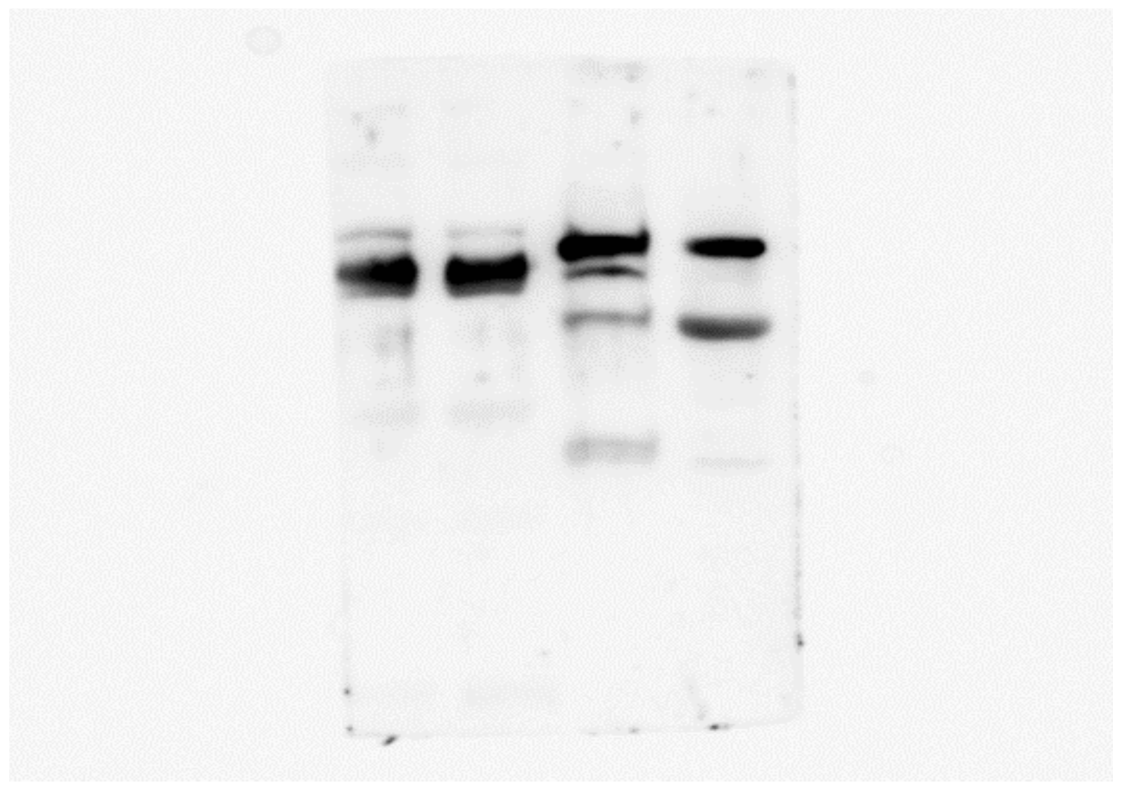

Supplement: Source data 1. — Zip folder containing raw and uncropped images for Western blots and Excel spreadsheets of quantitation. [file elife-72559-supp2.zip › 20210812_CRACR2A_SourceData/Figure 2D_CRACR2A.tif]

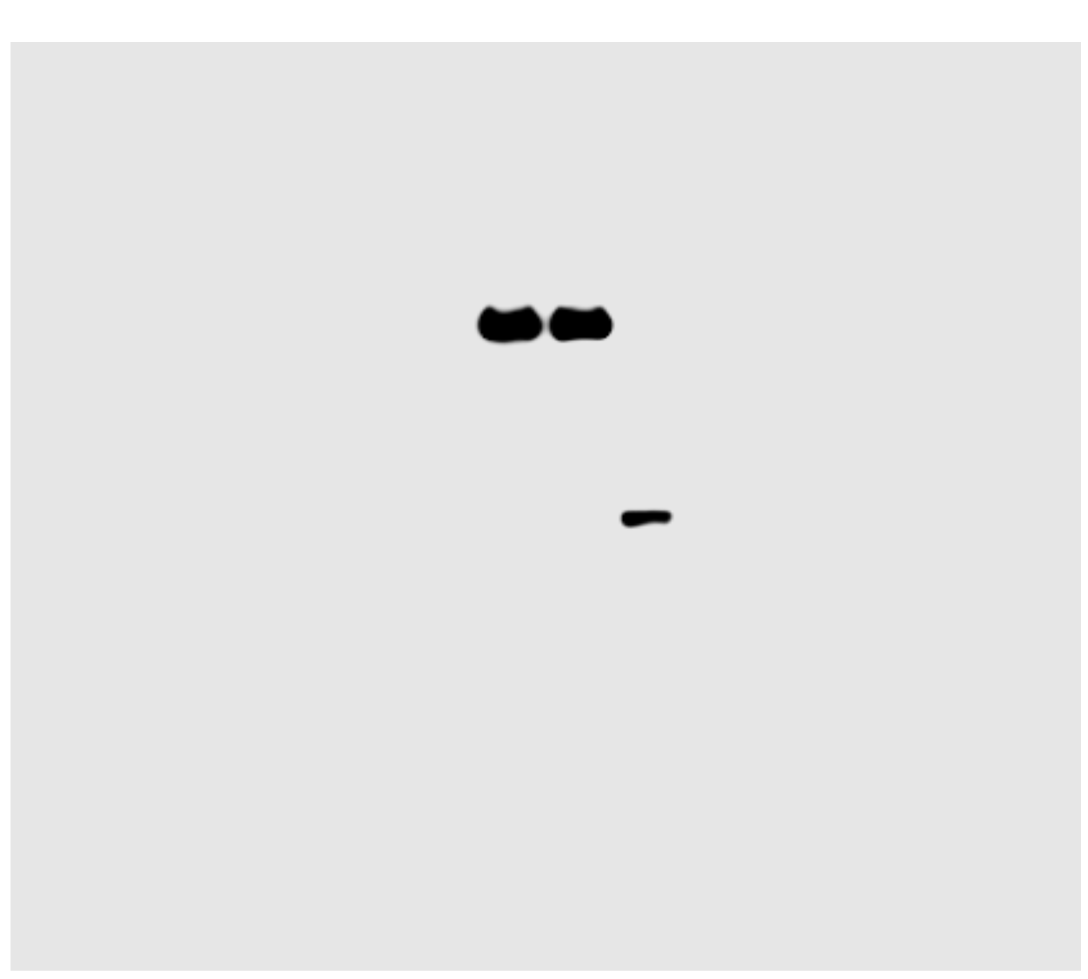

Supplement: Source data 1. — Zip folder containing raw and uncropped images for Western blots and Excel spreadsheets of quantitation. [file elife-72559-supp2.zip › 20210812_CRACR2A_SourceData/Figure 5_figure supplement_1C_Flag.tif]

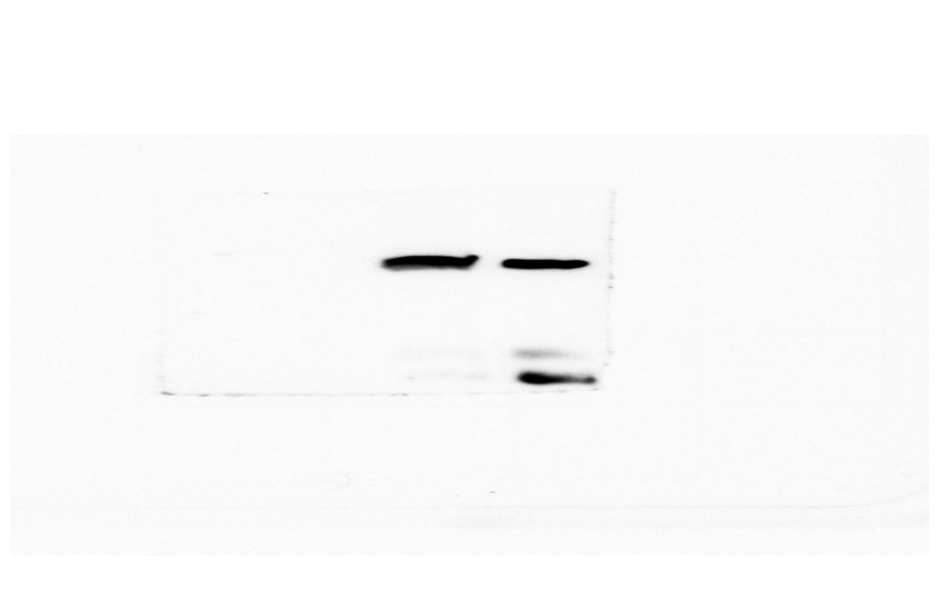

Supplement: Source data 1. — Zip folder containing raw and uncropped images for Western blots and Excel spreadsheets of quantitation. [file elife-72559-supp2.zip › 20210812_CRACR2A_SourceData/Figure 2D_actin.tif]

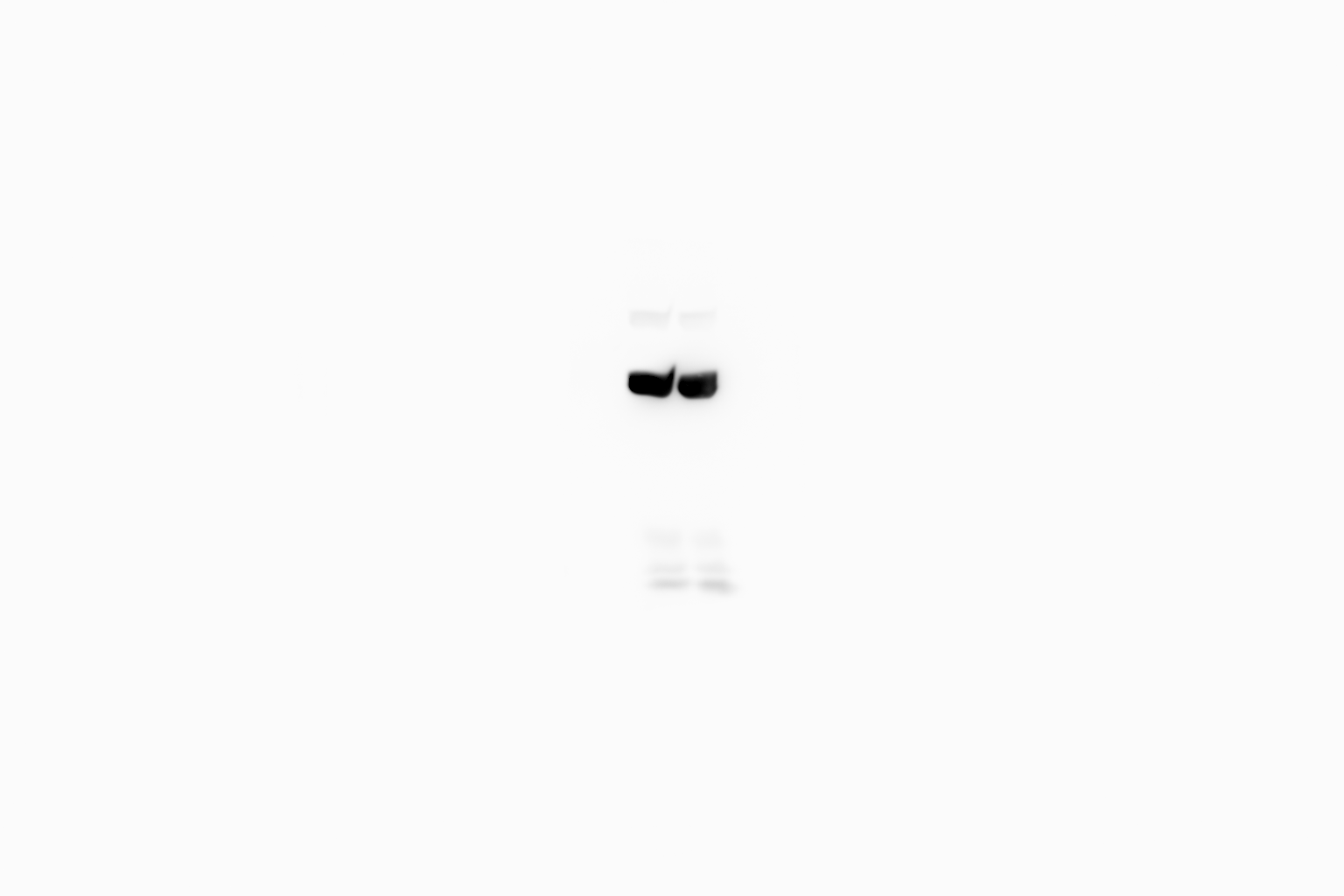

Supplement: Source data 1. — Zip folder containing raw and uncropped images for Western blots and Excel spreadsheets of quantitation. [file elife-72559-supp2.zip › 20210812_CRACR2A_SourceData/Figure 4_figure supplement 1_STIM1_ACTIN.tif]

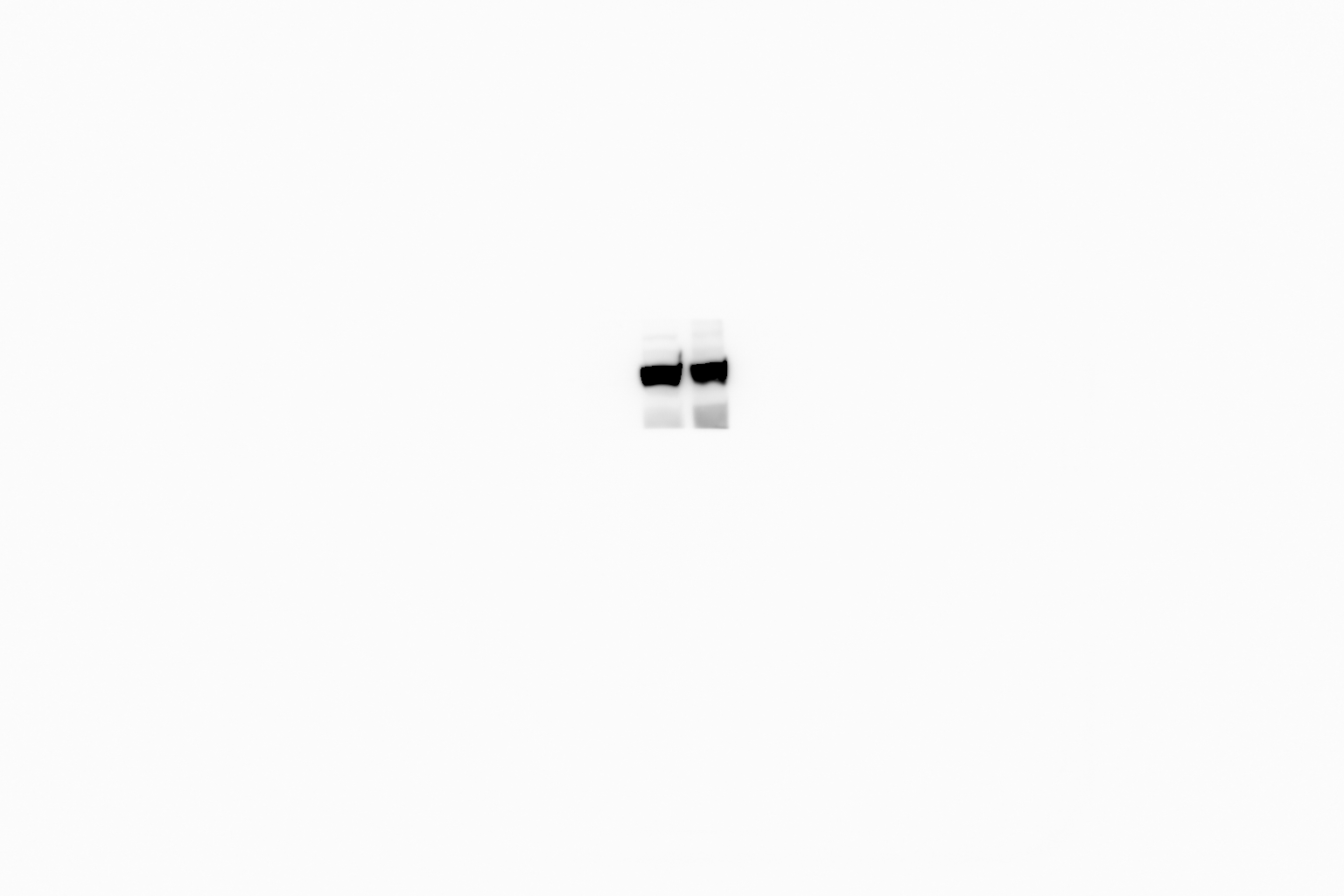

Supplement: Source data 1. — Zip folder containing raw and uncropped images for Western blots and Excel spreadsheets of quantitation. [file elife-72559-supp2.zip › 20210812_CRACR2A_SourceData/Figure 4_figure supplement 1_STIM1_WB.tif]

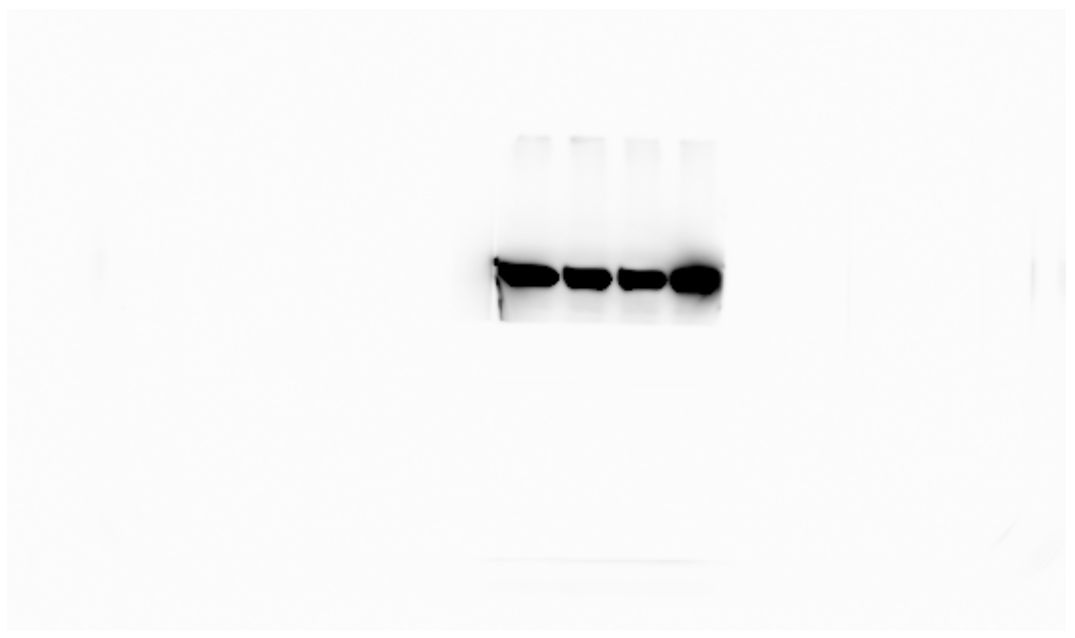

Supplement: Source data 1. — Zip folder containing raw and uncropped images for Western blots and Excel spreadsheets of quantitation. [file elife-72559-supp2.zip › 20210812_CRACR2A_SourceData/Figure 7A_Stim1-His_left.tif]

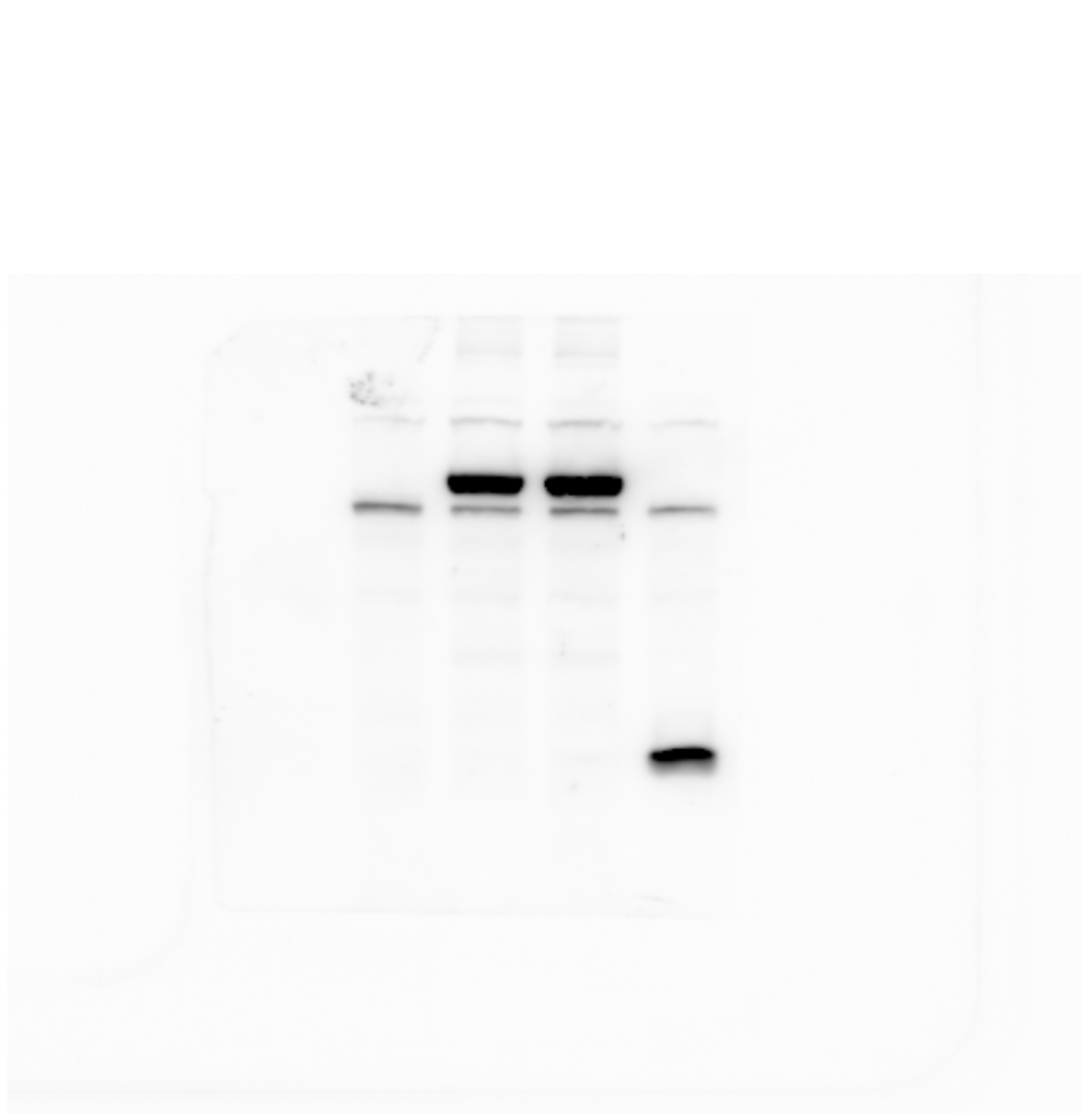

Supplement: Source data 1. — Zip folder containing raw and uncropped images for Western blots and Excel spreadsheets of quantitation. [file elife-72559-supp2.zip › 20210812_CRACR2A_SourceData/Figure 5_figure supplement 1_Flag.tif]

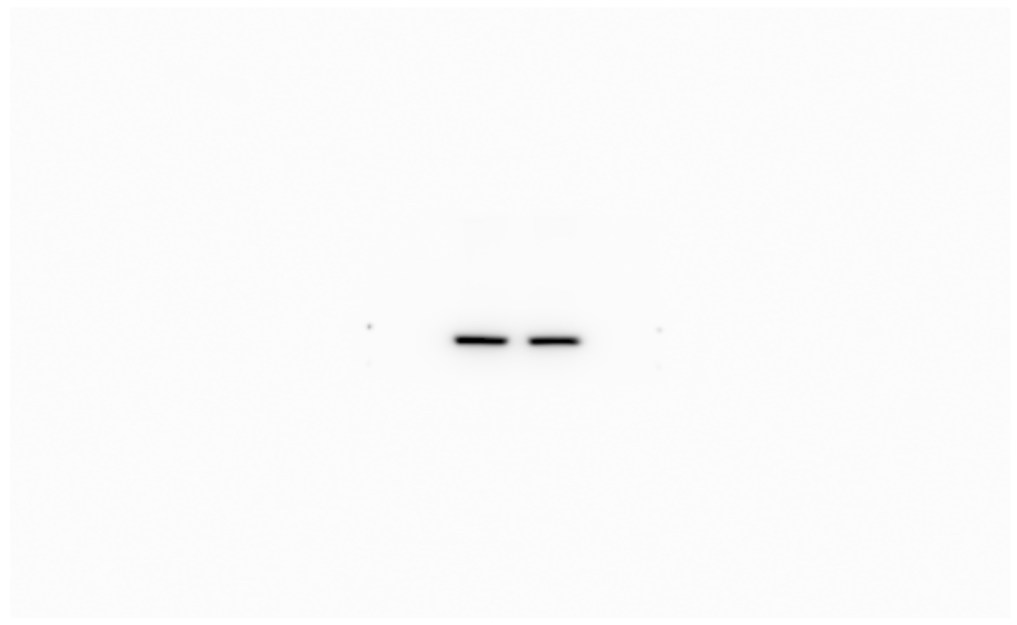

Supplement: Source data 1. — Zip folder containing raw and uncropped images for Western blots and Excel spreadsheets of quantitation. [file elife-72559-supp2.zip › 20210812_CRACR2A_SourceData/Figure 7A_Stim1-His_Right.tif]

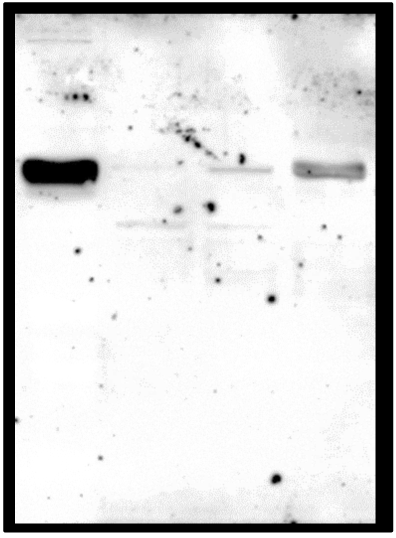

Supplement: Source data 1. — Zip folder containing raw and uncropped images for Western blots and Excel spreadsheets of quantitation. [file elife-72559-supp2.zip › 20210812_CRACR2A_SourceData/Figure 5_figure supplement _1_CRACR2A.tif]

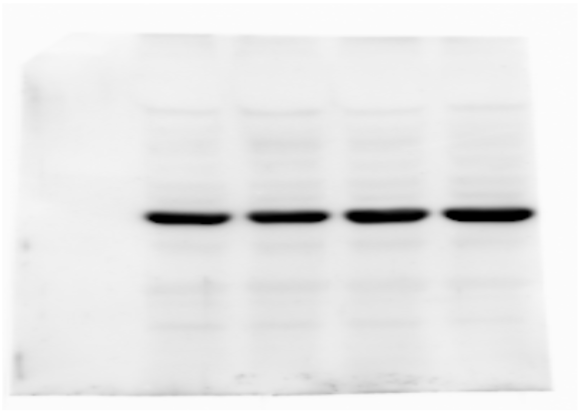

Supplement: Source data 1. — Zip folder containing raw and uncropped images for Western blots and Excel spreadsheets of quantitation. [file elife-72559-supp2.zip › 20210812_CRACR2A_SourceData/Figure 5_figure supplement 1B_actin.tif]
